# Implementation of a community health worker-focused team-based model of care: What modifications do clinics make?

**DOI:** 10.3389/frhs.2023.989157

**Published:** 2023-01-30

**Authors:** Laura J. Sotelo Guerra, Janette Ortiz, Kendra Liljenquist, Peter G. Szilagyi, Kevin Fiscella, Lorena Porras-Javier, Gina Johnson, Lisa Friesema, Tumaini R. Coker

**Affiliations:** ^1^ Center for Child Health, Behavior, and Development, Seattle Children's Research Institute, Seattle, WA, United States; ^2^ Department of Pediatrics, University of California, Los Angeles, CA, United States; ^3^ Department of Pediatrics, University of Washington School of Medicine, Seattle, WA, United States; ^4^ Department of Family Medicine, University of Rochester, Rochester, NY, United States; ^5^ Northeast Valley Health Corporation, San Fernando, CA, United States; ^6^ Community Health Care, Tacoma, WA, United States

**Keywords:** clinical practice redesign, adaptation, well-child care, preventive care, community health workers

## Abstract

**Background:**

Team-based care offers potential for integrating non-clinicians, such as community health workers (CHWs), into the primary care team to ensure that patients and families receive culturally relevant care to address their physical, social, and behavioral health and wellness needs. We describe how two federally qualified health center (FQHC) organizations adapted an evidence-based, team-based model of well-child care (WCC) designed to ensure that the parents of young children, aged 0–3, have their comprehensive preventive care needs met at WCC visits.

**Methods:**

Each FQHC formed a Project Working Group composed of clinicians, staff, and parents to determine what adaptations to make in the process of implementation of PARENT (Parent-Focused Redesign for Encounters, Newborns to Toddlers), a team-based care intervention that uses a CHW in the role of a preventive care coach. We use the Framework for Reporting Adaptations and Modifications to Evidence-based interventions (FRAME) to chronicle the various intervention modifications and the adaptation process, focusing on when and how modifications occurred, whether it was planned or unplanned, and the reasons and goals for the modification.

**Results:**

The Project Working Groups adapted several elements of the intervention in response to clinic priorities, workflow, staffing, space, and population need. Modifications were planned and proactive, and were made at the organization, clinic, and individual provider level. Modification decisions were made by the Project Working Group and operationalized by the Project Leadership Team. Examples of modifications include the following: (1) changing the parent coach educational requirement from a Master's degree to a bachelor's degree or equivalent experience to reflect the needs of the coach role; (2) the use of FQHC-specific templates for the coach's documentation of the pre-visit screening in the electronic health record; and (3) the use of electronic social needs referral tools to help the coach track and follow up on social need referrals. The modifications did not change the core elements (i.e., parent coach provision of preventive care services) or intervention goals.

**Conclusions:**

For clinics implementing team-based care interventions, the engagement of key clinical stakeholders early and often in the intervention adaptation and implementation process, and planning for intervention modifications at both at an organizational level and at a clinical level are critical for local implementation.

## Introduction

The National Academies of Science, Engineering and Medicine's consensus report, *Implementing High-Quality Primary Care*, defines high-quality care as delivered by interprofessional care teams that include individuals such as community health workers (CHWs) to ensure that patients receive whole-person integrated care (1). Evidence-based interventions that incorporate non-clinicians as part of a team-based approach to care have also been described as a key element in the provision of high-quality, equitable child preventive care, or “well-child care” (WCC) services (2–5). These “non-clinicians” can be CHWs, and more generally, are individuals who, in addition to the primary care clinician, provide direct education, guidance, or other preventive services.

WCC is a critical foundation of pediatric primary care in the United States; these visits comprise over one-third of all outpatient visits for infants and toddlers and provide a unique opportunity to address social, developmental, behavioral, and health issues (6, 7). The American Academy of Pediatrics (AAP), through its periodicity schedule, and Bright Futures Guidelines, recommends that children receive at least 10 WCC visits over the first 3 years of life, and that these visits include anticipatory guidance (counseling and education on a broad variety of topics), psychosocial and social needs screening, developmental surveillance, and screening (6, 8). Despite these clear and specific national guidelines for WCC, many families do not receive all recommended preventive services, including those most supported by the evidence and likely to improve health outcomes (9–13). Further, there is ample evidence that the delivery of WCC can be improved through clinical practice redesign interventions (3).

Parent-Focused Redesign for Encounters, Newborns to Toddlers (PARENT) is an evidence-based model of WCC that was previously developed for children aged 0–3 years in low-income families, to ensure that when families attend a WCC visit, their preventive care needs are met (14–16). PARENT is a team-based approach where a trained CHW in the role of a health educator (a “parent coach”) is added to the clinical care team and is responsible for providing anticipatory guidance, social needs screening and referral, and developmental or behavioral monitoring, screening, and guidance at each WCC visit. Integrating the parent coach into the clinical care team reduces the reliance on the physician as the sole provider of WCC services, while allowing families more time at visits to address their needs, in the context of a longitudinal relationship with an additional member of their preventive care team—the parent coach. As a trained CHW, the coach can enable the primary care team to provide more culturally relevant and family-centered preventive care. CHWs in pediatric primary care settings, when effectively integrated into clinical care teams, have been shown to improve health outcomes and address families' social determinants of health (17).

A randomized controlled trial of PARENT at two pediatric practices serving a low-income, predominately Latinx population demonstrated robust improvements in the receipt of WCC services, experiences of care, and reduced visits to the emergency department (ED) (15). It was unclear, however, whether this model for WCC delivery, developed and implemented in an academic-community partnered process by two independent, Medicaid-focused pediatric practices and one parent coach, could be adapted to other clinical settings.

For clinics and practices considering the implementation of PARENT, or other evidenced-based team approaches to WCC, the implementation process can seem daunting (18). Further, adaptations are necessary to fit the intervention to the contextual factors of each new practice, clinic, team, and community (19, 20). The aim of the present study was to examine the adaptation process in the implementation of PARENT, with three parent coaches, for five clinical sites in two multisite federally qualified health centers (FQHCs) as a new way of providing WCC for their population aged 0–3 years. Understanding how, when, and why the clinics made intervention adaptations during their implementation of the intervention will be important for clinics or practices to understand when considering moving to team-based primary care.

## Materials and methods

The adaptation and implementation process of PARENT was part of a cluster randomized controlled trial. Our clinical partners were two multisite FQHCs: one in Los Angeles County, California, with six participating clinical sites; and the other in Tacoma, Washington, with four participating clinical sites. Together, these 10 clinics serve a population of children that is approximately 95% Medicaid-insured and over 70% Latino. The clinics were randomized (stratified by organization and clinic size) to intervention or control. The study was approved by the Seattle Children's Hospital Institutional Review Board.

We used the Framework for Reporting Adaptations and Modifications-Enhanced (FRAME) to document modifications that occurred as a part of implementation of PARENT by these clinics (21). FRAME is used to describe modifications that occur in the implementation of evidence-based interventions; it consists of a series of questions to provide critical information of when and how modifications occurred, whether it was planned or unplanned, the relationship to fidelity, and the reasons and goals for modification. The FRAME uses both of the terms, modifications and adaptations, as defined in work by Stirman et al. (22, 23), with adaptations referring to “a process of thoughtful and deliberate alteration to the design or delivery of an intervention,” and modifications a more inclusive term for changes, whether proactive or in reaction to unanticipated challenges that arise during implementation or conduct of the intervention.

### Project Working Groups

Each FQHC created its own Project Working Group (PWG) to guide the adaptation and implementation of PARENT (see Table 1 for description of Project Working Group composition). The Project Working Group consists of clinic providers, staff, and clinical leaders, as well as parents who attend the clinic. Both Project Working Groups met eight times over a 12-month period (1 October 2017 to 30 September 2018) to adapt PARENT to meet the needs of their clinical sites and the families they served. Project Working Group meetings were facilitated by the study principal investigator (PI). Each Project Working Group meeting focused on specific elements of the intervention, and the Project Working Group’s task at each meeting was to determine how that intervention element would be implemented in their clinic sites, and what adaptations would be necessary (Table 2).

**Table 1:** Project Working Group description for each FQHC.

FQHC 1			FQHC 2	
Parents	Parent Representative	*n* = 5	Parent representative	*n* = 2
Medical team	Parent coaches	*n* = 2	Parent coach	*n* = 1
	Medical assistant	*n* = 6	Medical assistant	*n* = 2
	Nurse practitioner	*n* = 2	Registered nurse	*n* = 2
	Medical providers	*n* = 4	Medical providers	*n* = 3
Clinic Admin.	Information technology specialist	*n* = 1	Clinic manager	*n* = 4
	Clinic administrators	*n* = 6	EHR configuration analyst and coordinator	*n* = 2
	Public Health Program Manager	*n* = 1	Front desk staff	*n* = 2
Admin. Leadership	Administrator of Special Projects and Ancillary Services	*n* = 1	Quality improvement director	*n* = 1
	Director of Quality and Health Education	*n* = 1	Quality improvement manager	*n* = 1
	Medical Director/Pediatrician	*n* = 1	Medical director/pediatrician	*n* = 1

FQHC, federally qualified health center; EHR, electronic health record.

**Table 2:** Planned topics for Project Working Group meetings.

Month and year	Project Working Group topics discussed
October 2017	• Research team and clinic staff introductions • Project aims • Project Working Group operations • PARENT intervention: background and project plan • Visit shadowing: discussion
November 2017	• Parent coach role • Well-child care visit process • Well-child care visit workflow
December 2017	• Well-child care visit flow • Parent screening tools • Parent coach documentation
January 2018	• Parent coach documentation • Text message service
February 2018	• Parent pre-visit screening tools • Parent coach documentation • Text message service • Shadowing visit pilot testing
May 2018	• Mock of pre-visit questionnaires • Bright futures psycho-social recommendations • Parent coach template in the EHR (2-month)
June 2018	• Discussion of the next 12 months of study timeline • Discussion of intervention implementation and challenges • RCT participant recruitment procedure
October 2018	• Adaptation and implementation phase complete • Recruitment/enrollment • Data collection • Outcome measures
November 2019	• Revisit clinic workflow • Newborn and 2–4-week visit (EHR and pre-visit questionnaire) • Parent coach time tracking

EHR, electronic health record; PWG, Project Working Group; RCT, randomized controlled trial; PARENT, Parent-Focused Redesign for Encounters, Newborns to Toddlers.

### Academic Research Team

The Academic Research Team comprises the study PI, research coordinators, project manager, and co-investigators. Our Academic Research Team collected qualitative data through Project Working Group meeting recordings and notes on the intervention modifications that were made by the clinics as part of the adaptation and implementation process; all modifications were extracted from these qualitative data by the study PI and study staff, and discussed with the full Academic Research Team, after each Project Working Group meeting.

### Project Leadership Team

The Project Leadership Team consisted of the study Principal Investigator, project manager, and key clinical leaders at the FQHC. The purpose of the Project Leadership Team was to operationalize the modifications from the Project Working Group.

In the present study, we report these modifications that the clinic made during their adaptation and implementation process of the PARENT intervention. We used field notes collected during all project meetings and referred to meeting audio-recordings, when needed. From the notes, we compiled tables of changes that the project working group made; these were any alterations from the original pilot version of PARENT, in structure, content, or process. We utilize the FRAME as an organization of our results. The first two FRAME questions describe study methods and are described here; the remaining FRAME questions are answered in the “Results” section.

### The Project Working Group and process for modifications

The first of the FRAME questions focuses on when and how in the implementation process intervention were modifications made. Each PWG included clinical staff (clinician providers, medical assistants, nurses), clinic managers, parents of clinic patients, clinic front desk staff, and FQHC administrative leadership (medical director, quality director, and program manager for public health for FQHC 1, and the medical director and quality improvement manager for FQHC 2). Parent members were parents who had at least one child aged 12 months or older who received 0- to 3-year-old well-child care visits at that clinical site within the past year, and were nominated by the clinic staff or clinicians to the Project Working Group.

Parent Project Working Group members received an honorarium for their participation in the Project Working Group, as well as compensation for transportation and childcare. Per organizational policies, the FQHC personnel were not able to receive honoraria or compensation for their time on the Project Working Group, as all meetings occurred during their typical workday at the FQHCs. Research staff met with parents before and between Project Working Group meetings, as needed, to review PowerPoint presentations and planned Project Working Group discussion items. Of the seven parent members across both Project Working Groups, four preferred English language for communication and three preferred Spanish. A bilingual, native Spanish-speaker, research team member attended all Project Working Group meetings to provide a real-time interpretation for the Spanish-speaking parents; the Spanish-speaking parents were also provided with Spanish-language versions of the PowerPoint presentations and meeting handouts.

Each FQHC created a Project Leadership Team that met twice monthly, to guide the work of the Project Working Group. For both FQHCs, this team included the principal investigator, project manager, FQHC Medical Director, the Quality Director, Education Manager, and research coordinators.

The Project Working Group determined when a modification should be made and determined what that modification should be. The Project Leadership Team was often responsible for operationalizing the adaptation, and in some cases, the clinic team made additional modifications during intervention implementation at the clinical sites.

### The second FRAME question focuses on whether modifications were planned/proactive or unplanned/reactive

The modification of PARENT was planned and proactive. As part of the implementation process, the Project Working Groups were tasked with determining what modifications would be necessary for successful implementation at their clinical site.

## Results

We present the findings of the remaining FRAME questions.

### FRAME question 3: Who determined that the modification should be made?

At the first Project Working Group meeting, the groups determined that all participants in the Project Working Group held an equal stake in the decision-making process and decisions were made by consensus. At every Project Working Group meeting, parent perspectives were explicitly elicited before any group decision. In addition to the FQHC-level Project Working Group meetings, we held clinic site-specific meetings to gather feedback from clinic personnel who were not part of the Project Working Group. The feedback from these conversations was brought back to the Project Working Group and discussed to establish a broader consensus and determine the best application for each intervention site.

### FRAME question 4: What was modified?

One of the first modifications was to focus the implementation on children aged 0–2 years, rather than 0–3 years as the original intervention did. The Project Working Group made this modification because it was clear that the evaluation of the intervention in their sites would focus on children aged 0–2 years, and they wanted the implementation to cover this same age range to optimize the intervention for this group. Other modifications are described below and in Table 3.

**Table 3:** Examples of intervention modifications by the PWG task.

	Level of modification	Type of modification	Contextual factors
PWG Meeting Task 1: *Process and Documentation*—Establish and determine content, periodicity (timing of delivery), format, and procedures for the pre-visit screening tool			
The pre-visit screening tool was changed from a web-based questionnaire with pdf of results uploaded to the EHR, to using the AAP's BFPQ (24, 25) by paper, or through EHR templates due to concerns of cost and sustainability of the web-based tool	Organization	Change in materials/tools	Sustainability and cost
Added more social needs screening content to the BFPQ	Organization	Added content	Patient and family needs
Created an FQHC-specific screening periodicity to reduce parent burden	Organization	Periodicity change	Patient and family needs
PWG Meeting Task 2: *Documentation*—Generate documentation system for parent coach complementing current EHR system			
EHR specialists for each FQHC created new templates for documentation of the pre-visit screening in the EHR	Organization	Change in materials/tools	Clinic efficiency
PWG Meeting Task 3: *Workflow*—Develop a clinical workflow to support the PARENT intervention			
Clinic site-specific workflow was created that varied by clinic staffing, size, and provider type (i.e., family medicine or pediatrics) and provider preference	Clinic	Process change	Clinic efficiency
Timing in clinic workflow for parent coach discussion modified to account for availability of exam rooms	Provider	Process change	Clinic efficiency
Electronic social needs referral sources were added to help coach track and follow up on social need referrals	Organization	Change in materials/tools	Clinic efficiency patient and family needs
PWG Meeting Task 4: *Training*—Prioritize hiring and training priorities for the parent coach			
Modified educational requirement from Master's degree to bachelor's degree or equivalent experience to reflect needs of coach role	Organization	Structural change	Sustainability and cost Patient and family needs
PWG Meeting Task 5: *Communication*—Determine methods and processes for communication and coordination of information sharing among WCC team			
Format of warm hand-off modified based on clinic capacity and provider practice style (through EHR or in person)	Provider	Process change	Clinic efficiency
PWG Meeting Task 6: *Workflow*—Outline the key implementation processes for the intervention			
Additional workflow adjustments made at the clinic level and provider during “run-in” period, to address newly identified workflow challenges	Provider	Process change	Clinic efficiency

AAP, American Academy of Pediatricians; BFPQ, Bright Future Pre-visit Questionnaire; EHR, electronic health record; FQHC, federally qualified health center; PWG, Project Working Group; WCC, well-child care.

#### Pre-visit screening tool

In the original intervention, the parent coach used a web-based pre-visit parent questionnaire to guide the content of the well-child care visit and identify areas of need and parent priorities for the visit. Both Project Working Groups decided to replace this web-based parent questionnaire with the print version of the AAP’s Bright Futures Pre-visit Questionnaire (BFPQ) (6, 24, 25) to avoid costs that would be required to maintain and link the web-based tool to their electronic health record (EHR). Each Project Working Group made modifications to the BFPQ, included adding social needs screening items, and omitting developmental milestone surveillance items for visits that already included a standardized developmental screening tool (8).

#### Parent coach EHR documentation

In the original intervention, the pre-visit questionnaire responses were uploaded as a .pdf file in the EHR, and the coach documented their visit, using “dot phrases” (an EHR keyboard shortcut to insert pre-determined text) in the notes section of the encounter in the EHR. Both Project Working Groups decided to work with their EHR specialists to create BFPQ and coach EHR templates for documentation. Since the two FQHCs had the same EHR systems, they shared information on creating the templates; each WCC visit (newborn to 24 months) had a FQHC-specific, modified BFPQ embedded in the parent coach EHR workflow. Although the templates to create the EHR-embedded BFPQ were similar across FQHCs, there were key difference in both the content and the places in the EHR encounter where the adapted BFPQ responses appeared. This adaptation allowed for documentation standardization in the EHR among each WCC type (e.g., newborn visit, 2-month visit, etc.) and across each clinic site and FQHC, thereby reducing error in repeatability of task, and consistency of care, while allowing for customization for each site.

#### Clinic workflow

The clinical well-child care teams (provider, medical assistant, clinic manager, and coach) created the first iteration of the clinic workflow, which was revised over multiple Project Working Group meetings and Project Leadership Team meetings. Each FQHC had its own clinical workflow to accommodate PARENT. Once the parent coaches began work in the clinic, and additional workflow needs were identified by the clinical team, the workflow was revised again with additional clinic-level changes to meet the unique needs of each of the five intervention clinical sites. In addition to the workflow being the explicit focus of three Project Working Group meetings, we allotted time at all Project Working Group meetings, as well as at clinic site-specific meetings, to discuss the workflow challenges that emerged. The full Project Working Group provided input for that specific workflow challenge, which included the parents’ view of the impact on their visit experience.

The two Project Working Groups also decided to adopt different systems for the parent coach to track social needs referrals, based on tools in other areas of the FQHC (e.g., adult primary care). FQHC 1 utilized One Degree (https://www.1degree.org/), a web-based tool, to help the parent coaches track patient and family use of community referrals, while FQHC 2 utilized a database created by the Department of Public Health to help the parent coach track the family connection with community referrals.

#### Parent coach hiring and training

The FQHCs used their standard hiring process to hire the parent coaches. The parent coach salary during the study period was funded by the project grant. The Project Working Group determined their parent coach did not necessarily need a Master's degree to provide these services (as the coach in the original project did). Specifically, the Project Working Group was concerned that requiring a Master's degree would eliminate potential candidates from diverse backgrounds who would have been a good fit for the role. The Project Working Group and Project Leadership Team determined that the coach would be required to have either a bachelor's degree, or alternatively, could have equivalent experience in child development, health education, early childhood, or related fields, and that the coach should also be bilingual in English and Spanish to meet the language needs of their population. The Project Working Group prioritized the qualities of shared lived experiences with families, connection to community served by the clinic, and ability to engage with and partner with families as some of the key qualities of the parent coach. These qualities match the qualities of CHWs, defined by the Centers for Disease Control and Prevention as “a frontline public health worker who is a trusted member of a community or who has a thorough understanding of the community being served” (26).

The original parent coach training curriculum was utilized, which consisted of didactics and observed, mock, and precepted visits with real-time feedback and coaching from a trainer and/or member of the well-child care team. The training was inclusive of broad core competencies for CHWs (27) (e.g., Professional Conduct, Communication, Outreach, Knowledge Base) and, more specifically, included interactive modules on Community Resource Building, Trauma-informed Care, Motivational Interviewing, Social Needs, Child Development, and Bright Futures Preventive Care Guidelines (6), as well as other topics. The Project Working Group added additional elements to the coach training, such as positive parenting training techniques; these elements were added to the training for all three coaches.

#### Well-child care team communication and coordination

The five intervention clinics for the FQHCs differed enough in physical size (number of exam rooms), volume of patients, and number of clinicians seeing 0–24-month WCC visits, that each clinical site needed a different method for parent coach–WCC team communication and hand-off between the parent coach and clinician. One clinic used a verbal hand-off for most visits, while another utilized the documentation in the EHR to share information for the visit “hand-off.” Still, in another clinical site in which there were sometimes not enough exam rooms to accommodate a parent coach, the workflow for the parent coach was to call the parent the day before the visit to provide WCC services *via* phone, and later do a quick check-in with them in person on the day of the visit. In another clinical site that utilized family medicine physicians as well-child care providers, the volume of 0–24-month visits was too low to account for a full day of parent coach time at the clinic, so the coach was trained, at the clinic, to provide preventive services for school-aged children as well (e.g., nutrition counseling, social needs screening, and referrals). During the Project Working Group discussion on communication and coordination for the parent coach role, clinicians and staff were able to fully describe what would be logistically feasible at their clinical site. The parent Project Working Group members responded with how any proposed workflow might impact their visit experience.

The Project Working Group also discussed how to account for potential challenges in the family–well-child care team dynamic and relationship. As an example, one provider was concerned that the parent coach completed screening and referral for social needs before she, as the provider, saw the family for her part of the visit. Thus, her concern was that the parent might perceive her as not caring about that social need. The Project Working Group, including the parent members of the Project Working Group, discussed potential ways to avoid this type of unintended consequence, including having the provider give a quick summary of what the parent coach accomplished during their part of the visit, so that the parent would understand that they (provider and parent coach) were a part of the same team and working together.

### FRAME question 5: At what level of the delivery was the modification made?

The modifications described above span various levels, including the FQHC organization level, the clinic level, and individual provider level.

### FRAME question 6: What was the type or nature of context- or content-level modifications?

The nature of the modifications varied as well, and included adding elements, tailoring, changes in materials, and reordering elements of the intervention.

### FRAME question 7: To what extent were the modifications fidelity consistent?

Whether the modification of the intervention is fidelity consistent refers to the extent that the core elements of the intervention were retained. In Table 4, we outline the core elements of PARENT, and to the extent that the modifications kept these core elements in place, the modifications were deemed to be “fidelity consistent.” The modifications did not change the core elements (i.e., parent coach provision of preventive care services) or goals of the intervention (i.e., ensuring that low-income families have their preventive care needs met). The Project Working Group did not have any *a priori* restrictions on elements of the intervention that could be modified but they did maintain its key elements for a fidelity-consistent intervention.

**Table 4:** Description of the PARENT intervention.

Overview	The coach provides anticipatory guidance, psychosocial and social needs screening and referral, and developmental/behavioral surveillance, screening, and guidance at each well-visit. The coach uses pre-visit screening to customize the visit to the parents' needs, and an automated text message service to reinforce the periodic, age-specific health messages provided to families at well-visits. The PARENT well-visit includes time with the pediatric clinician, focused on topics requiring their expertise.
Intervention core elements	
Parent coach	The coach receives training to provide comprehensive WCC services. The coach role is similar to a health coach, health educator, or wellness coach, and is generally a health educator, care coordinator, or advanced medical assistant who has received upwards of 80 h of training consisting of self-directed learning based on Bright Futures, relationship building with community organizations near each clinical site, mock visits, and preceptor-observed visits at the practices.
Pre-visit screening	The **Well Visit Planner**, developed by the CAHMI is a pre-visit tool that asks parents a set of standardized, pre-visit questions anchored to Bright Futures guidelines and tailored for each well childcare visit. Once completed, the responses are uploaded along with a .pdf file of the completed questionnaire in the open WCC encounter in the EHR.
Text messages	Bright Futures-based text messages are adapted to meet the needs of the practices. Parents receive a welcome message from their practice, with subsequent weekly to bi-weekly age-specific messages delivered throughout the intervention. Messages focus on age-appropriate anticipatory guidance, health education, developmental tips, parent resources, and reminders. Most messages include a link to an educational website (e.g., healthychildren.org) with video and written information on that specific topic or include the practice's phone number for visit scheduling.
Visit process	Upon arrival, the child is registered, weighed and measured by the medical assistant, and roomed (usual care process). The coach enters the patient room and uses data from the Well Visit Planner to: 1) discuss parent-identified concerns (anticipatory guidance topics), 2) review any additional concerns highlighted by the pre-visit tool, and 3) conduct developmental surveillance, address any developmental or behavioral concerns, and assist the parent in completing a standardized screening instrument for developmental delay or autism (at AAP-recommended visits). If the pre-visit tool results indicate a need for community referrals for social needs, the coach provides these referrals at this time. The coach documents the visit with the family in the EHR, highlighting results of developmental/behavioral screening and any issues that the clinician needs to review at the top of the encounter page. The coach spends 15–20 min with the family, based on the parents’ needs. When necessary, the coach can conduct their portion of the visit virtually.

AAP, American Academy of Pediatricians; EHR, electronic health record; PARENT, Parent-Focused Redesign for Encounters, Newborns to Toddlers; WCC, well-child care; CAHMI, Child and Adolescent Health Measurement Initiative.

### FRAME question 8: What were the reasons for the modifications? The intent or goal of the modification? Contextual factors that influenced the decision?

The reasons, intent, or goals for the modification, and contextual factors that influenced the decisions for the modifications, are variable and described above. We also provide various contextual factors for the modifications described in Table 3. The contextual factors range from the organization's need to consider sustainability and cost, or patient and family needs, to overall clinic efficiency.

## Discussion

Using FRAME, we describe the adaptations that five clinical sites made to an evidence-based, team-based approach to preventive care visits, for clinic-level implementation. The clinic sites implemented PARENT, a team-based approach to well-child care that utilize a “parent coach” for the delivery of routine preventive care services for children aged 0–3 years and their families. Engaging a Project Working Group, which represented the FQHC organizational stakeholders, clinicians and staff, and parents, was critical for allowing the implementation of the team-based model of care to have a clear framework that included a consistent team structure and clinical processes, while still allowing each clinical site to make necessary adaptations at the clinic level. Each FQHC organization, and its individual clinical sites, needed to make its own adaptations to PARENT, and these adaptations created a team-based model of care that had some key differences from the model of care that was originally designed and evaluated for effectiveness (15), while retaining the core elements of the PARENT. Intervention adaptations were made at various levels of delivery (FQHC organization, clinic, and individual provider), represented multiple types of changes and additions (e.g., changes in content, processes, materials/tools, and content), and decisions-making factors (e.g., a need for sustainability, or a focus on patient and family-centeredness, or clinic efficiency).

Local adaptations to evidence-based interventions have been described as changes that are made when evidence-based interventions are implemented in community settings (28). While deviations from evidence-based interventions can be seen as a threat to intervention fidelity, it is also important to recognize that local adaptation can lead to greater clinic-level engagement with the intervention, improved program outcomes, and thus intervention effectiveness (29). Barrera et al. recommend that further intervention evaluation occurs after local adaptation, and that interventions clearly identify, *a priori*, the core elements that are critical for fidelity so that community implementers know what can be adapted and what should not (28).

To both ensure fidelity to the core components of PARENT, as well as to the clinic-level adaptations of PARENT, the Project Working Group, Project Leadership Team, and Academic Research Team jointly created a process for checking the fidelity of the intervention at regular intervals. These fidelity checks were instituted to ensure that the PARENT intervention was being implemented and maintained as determined by the workflow created by the Project Working Group and approved by the Project Leadership Team, and iteratively revised by the clinic team. In this way the project, sought to optimize the autonomy of each Project Working Group in adapting the PARENT model within the constraints of its core elements while simultaneously ensuring fidelity. This is analogous to the concept of “paradoxical leadership,” where groups have goal clarity but also latitude in determining how to do their work (21, 22). The fidelity data will be an important element in our reporting of trial results and may also inform future implementations of PARENT.

Our findings also have important implications for the integration of CHWs in primary care teams. Multiple authors have emphasized the importance of effectively integrating CHWs into care teams, to reach the full potential of the CHW model of team-based care (17, 30). Our findings indicate that healthcare organizations and clinics that employ CHWs may need to make local adaptations to achieve an optimal level of clinic integration. The Community Health Worker Core Consensus Project (27) provides a useful resource that defines various CHW roles, skills, and qualities, including many that define the work of the parent coaches in this intervention (e.g., the role of providing culturally appropriate health education and information, and training in communication skills and for knowledge base), and can serve as a useful starting point for clinics aiming to integrate CHWs into a team-based approach to care.

While a team-based approach to care is recommended broadly for the delivery of equitable, high-quality primary care, each clinical setting and community will have its own needs for adapting evidence-based interventions for team-based care. Thus, a key limitation of our findings is that these adaptations may not be generalizable to other clinic settings implementing team-based well-child care. However, the process has potential generalizability. Specifically, the process of adaptation, using the principles of paradoxical leadership that offer multidisciplinary teams bounded latitude in adapting an intervention to their own setting, provides a template for adaptation while also informing an understanding of how these decisions were made, why, and by whom. This can serve as a guide for other clinical settings considering a shift toward team-based WCC.

It is not clear whether these modifications to PARENT will lead to positive outcomes for patients and families—that will be determined by the planned cluster randomized controlled trial. However, from the findings presented here, it is clear that ongoing improvement of the intervention relied on the diverse and multidisciplinary work group that included key representation of frontline staff, clinicians, administrators, and families in the adaptation process. This “Project Working Group” structure can be applied to intervention adaptation and also allows the preservation of the core elements and goals of an intervention. FRAME provides a useful structure for not only reporting, but in planning of intervention adaptations and modifications. Interventions for pediatric clinical delivery, particularly those focused on underserved communities, should engage clinical stakeholders early in the adaptation and implementation process and throughout, integrate parents fully in the process, and recognize that critical adaptations at multiple levels of care delivery will be necessary and beneficial to local implementation.

## Data Availability

The raw data supporting the conclusions of this article will be made available by the authors, without undue reservation.
